# 2-deoxy-2-[18]fluoro-D-glucose PET/CT (18FDG PET/CT) may not be a viable biomarker in Pompe disease

**DOI:** 10.1186/s40246-018-0145-7

**Published:** 2018-03-09

**Authors:** U. Plöckinger, V. Prasad, A. Ziagaki, N. Tiling, A. Poellinger

**Affiliations:** 1 Kompetenzzentrum Seltene Stoffwechselkrankheiten, Interdisziplinäres Stoffwechsel-Centrum: Endokrinologie, Diabetes und Stoffwechsel, Charité Universitätsmedizin Berlin, Augustenburger Platz 1, Campus Virchow-Klinikum, 13352 Berlin, Germany; 2 Department of Nuclear Medicine, Charité Universitätsmedizin Berlin, Berlin, Germany; 3 Department of Nuclear Medicine Universitätsklinik Ulm, Ulm, Germany; 4 Department of Diagnostic, Interventional and Pediatric Radiology, Inselspital Bern University Hospital, University of Bern, Bern, Switzerland

**Keywords:** Pompe disease, 2-deoxy-2-[18]fluoro-D-glucose PET/CT, Biomarker, MRI

## Abstract

**Background:**

Pompe disease (PD) is an autosomal recessive, lysosomal storage disease due to a mutation of the acid α-glucosidase (*GAA*) gene. In adult patients, PD is characterized by slowly progressive limb-girdle and trunk myopathy and restrictive respiratory insufficiency. Enzyme replacement therapy (ERT) is available, improving or stabilizing muscle-function in some and slowing deterioration in other patients. Unfortunately, there is no biomarker available to indicate therapeutic efficacy and/or disease activity. Whole body MRI depicts all skeletal muscles demonstrating foci of atrophic muscles, i.e., late and irreversible pathological changes. Any method indicating the localizations of increased muscle glycogen storage, muscle inflammation and/or degradation could possibly help identifying newly afflicted tissue and may be of prognostic value. We therefore investigated 2-deoxy-2-[18]fluoro-D-glucose (FDG) PET, a biomarker for glucose-metabolism, as a tool to evaluate disease activity and prognosis in PD.

**Methods:**

In a pilot study, we investigated four patients by FDG dynamic PET/CT while on ERT. One patient had FDG-PET/CT twice, before and after 12 months on ERT. Dynamic FDG-PET/CT quantifies the metabolic rate of glucose utilisation in mg/ml/min. MRI was performed in parallel with pelvic and thigh muscles semi-quantitatively scored for atrophy and disease-activity.

**Results:**

None of the muscles analysed showed a focally increased FDG-uptake. Thus, quantification of muscle glucose metabolism could not be calculated. However, increased FDG-uptake, i.e., increased glucose utilisation, was observed in the respiratory muscles of one patient with severe, restrictive respiratory failure. In contrast, specific MRI sequences showed oedematous as well as atrophic muscle areas in PD.

**Conclusions:**

Our pilot study demonstrates that FDG-uptake does not correlate with glycogen storage in vivo. In contrast, MRI is an excellent tool to demonstrate the extent of muscle involvement. Specific MRI sequences may even demonstrate early changes possibly allowing prognostic predictions or localization of early stages of PD.

## Background

Pompe disease (OMIM 232300) or glycogenosis type 2 is a lysosomal storage disease characterized by a mutation of the acid α-glucosidase (*GAA*) gene. Pompe disease (PD) is inherited in an autosomal recessive mode. Manifestations of PD in adult patients are a myopathy of limb-girdle and trunk muscles, with restrictive respiratory insufficiency as a possible complication. The disease is chronic and slowly progressive with some patients becoming wheel-chair-bound and respirator-dependent. The available enzyme replacement therapy effectively improves or stabilizes muscle function in some and slows deterioration of the disease in other patients [1, 2].

The phenotype/genotype correlation is poor and many patients harbour private mutations. Thus, genotyping is unable to predict the clinical course of an individual patient [3]. Clinical monitoring, i.e., follow-up of the individual patient, relies mainly either on semi-quantitative analysis of muscle function, like 6-min walk test, quick motor function test [4] or magnetic resonance tomographic imaging (MRI) of limb-girdle, paravertebral and thighs musculature and sometimes proximal arm musculature, or in rare cases, repeated muscle biopsies. Respiratory function is evaluated by lung function testing [forced expiratory vital capacity (FVC) and vital capacity (VC)], both while seated and supine. However, these clinical evaluations are flawed by a high intra- and inter-observer variation, as is the case for functional testing [6-min walk test [5, 6] and quick motor function test [4]. In addition, the test-results vary depending on the patient’s sex, age and day-to-day state of health. A reliable positive predictive result is therefore difficult to achieve and intra-individual variability is high. Due to the lack of more sensitive and accurate measurements, the 6-min walk test is defined as the gold-standard and has been used in clinical studies for the evaluation of motor function in adult patients with PD [3, 7].

Biochemical parameters, i.e., muscle enzymes like aspartate aminotransferase/glutamat-pyruvat-transaminase (AST/GOT), alanine aminotransferase/glutamat-oxalacetat-transaminase (ALT/GPT), creatinine-kinase (CK) or lactate dehydrogenase (LDH) are non-specifically increased in PD and fail to correlate with disease severity or activity. The glucose tetra-saccharide, Glcalpha1-6Glcalpha1-4glcalpha1-4Glc (Glc(4)), is a glycogen-derived limit dextrin that correlates with the extent of glycogen accumulation in skeletal muscle. A retrospective analysis of clinical records of 208 patients evaluated for PD by this approach showed Glc(4) having 94% sensitivity and 84% specificity for the diagnosis of PD. However, while indicating the overall disease burden, Glc(4) provided no information on the location and distribution of excess glycogen accumulation [8]. In addition Glc(4) excretion in adult-onset PD is low and ERT-induced declines may be very small [8].

MRI imaging demonstrates the distribution pattern of muscular atrophy, compensatory muscular hypertrophy and vacuolised muscle fibres. A study with 34 adult-onset PD patients compared whole body muscle MRI using T1w and 3-point Dixon imaging of thighs and the lower trunk region to a wide range of functional scales. This comparison demonstrated a strong correlation between muscle strength, muscle functional scales and the degree of muscle fatty replacement in muscle MRI. In addition, muscle MRI detected mild degree of fatty replacement in para-spinal muscles in pre-symptomatic patients [9]. Others used either quantitative proton-density fat-fraction (PDFF) whole-body MRI or quantitative MRI in late-onset PD and compared the results with manual muscle testing. According to their results, MRI was more sensitive than physical examination for detection of abnormalities in multiple muscle groups [10, 11]. Yet, the rather slow disease progression in PD is one difficulty for MRI monitoring of muscles changes. A retrospective study by Carlier et al. using quantitative MRI demonstrated muscle fatty infiltration increase on average by 0.9%/year, with the hamstring and adductor muscles showing the fastest degradation. Muscle water T2 mapping revealed that 32% of all muscles had abnormally high T2 in at least one of two successive examinations. When muscle water T2 was abnormal, fatty degenerative changes increased by 0.61%/year. Enzyme replacement therapy resulted in 0.68%/year slow-down of the muscle fatty infiltration, in both muscles with normal and high T2s [12]. Unfortunately, most MRI findings all indicate a rather late event in the course of the disease, i.e., muscle atrophy. Furthermore, an overall slow-down of muscle fatty infiltration of 0.68%/year may not allow an individual prognosis, neither on the natural course of the disease nor on the effectiveness of enzyme replacement therapy. In addition, MRI imaging poses a burden on patients with claustrophobia, is difficult to perform in patients with respiratory insufficiency in the supine position and can be painful due to the long duration of laying still on a hard bench.

Muscle biopsies using ultrastructural examination of the tissue demonstrated reduced lysosomal glycogen after 6 months of ERT, consistent with stabilization of the disease that was not represented in the MRI images acquired parallel to the biopsies [13]. However, muscle biopsies are fraught due to (i) changing tissue localization at repeated biopsies and (ii) the invasiveness of the procedure.

Thus, to date, none of the aforementioned methods allow for a simple, reproducible measurement of disease activity, localization of active disease and monitoring of therapeutic efficacy.

**Fig. 1:**
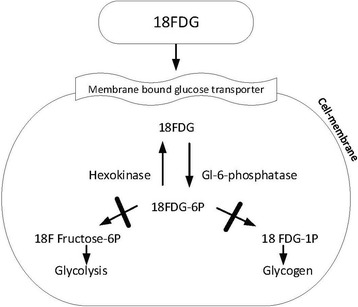
Scheme delineating cellular uptake of FDG

Glucose and/or FDG uptake by the cell is a receptor-mediated process. Glucose binds to the membrane bound glucose-transporter (GLUT), as does FDG. The receptor-ligand complex is taken up into the cell by endocytosis. There the receptor separates from its substrate in early endosomes and is passed on to recycling endosomes and, subsequently, stored in the GLUT-containing vesicular compartment. Once inside the cell, FDG is phosphorylated to FDG-6P, which cannot enter the glycolytic pathways and is therefore trapped within the cell (Fig. 1).

The mutation of acid α-glucosidase (*GAA*) gene leads to reduced concentration/activity of the enzyme. This results in a defective metabolism of lysosomal glycogen with tissue-specific glycogen accumulation. We hypothesized that the uptake of 2-deoxy-2-[18]fluoro-D-glucose should lead to an accumulation of the labelled glucose molecule in cells with pathological glycogen storage. The extent of accumulation would indicate the extent of glycogen storage, correlating with disease activity and possible therapeutic efficacy. The method would therefore allow for early detection of active foci of glycogen deposits, well before fat accumulation or muscle atrophy occurs.

We therefore undertook an explorative study investigating the usefulness of dynamic FDG-PET as an indicator of disease activity in late-onset PD and compared these results to routinely performed quantitative whole-body muscle MRI.

## Methods

We performed five FDG dynamic PET/CTs in four patients with late onset PD. In one patient, FDG-PET/CT was performed twice, before and 1 year after the initiation of ERT, while in all other patients FDG-PET/CT took place during ongoing ERT. Patient characteristics are given in Table 1.

**Table 1:** Patients characteristics

Patient number	1	2	3	4
Age (years)	51	59	27	45
Years of ERT	1.5	6.5	7	0
Sex	M	M	M	M
Age at diagnosis (years)	50	45	20	44
Mutation	c.(336-13 T > G); (1927G > A)	IVC-45 T > G; 536bpDel (IVS16 + 102_IVS17 + 31)	c.32-13 T > G; c.1128_1129delinsC	c-32-13 T > G; c.2214G > A
6-min walk test (m)	484	204	201	510
Wheelchair bound	No	No	No	No
Respiratory failure	Yes	Yes	Yes	No

^a^*SD* standard deviation

*FDG-PET Acquisition* Dynamic FDG-PET allows to quantify the metabolic rate of utilisation of glucose in mg/ml/min. Patients were kept fasting for more than 6 h, and serum glucose was measured prior to the injection of FDG. Patients were given 350 MBq of FDG as slow bolus over 20 s, and scanning was performed with the patients supine. Dynamic images were acquired over 60 min in list-mode immediately after the injection of the tracer. Images were reconstructed as follows: 3 × 20 s, 8 × 30 s, 5 × 1 min, 5 × 2 min, 4 × 5 min and 2 × 10 min. After the dynamic images, whole body images were acquired from base of skull to mid-thigh with 1.5 min/bed position. Lean body mass maximum standardized uptake value (SUVmax) was calculated for involved muscles; region of interest was drawn on the muscles showing hyperintensity on MRI. In addition, background activity was measured on the subcutaneous fat at the level of gluteus muscle as well as over the left ventricular cavity. For correction of the PET images, we used a CT attenuation correction map.

**Fig. 2:**
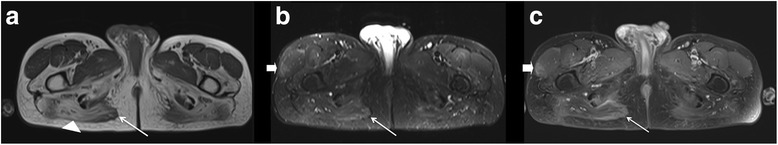
MR scans of a Pompe patient at the level just below the pelvic floor. There are different stages of muscular inflammation, or atrophy respectively, visible: T1w images show fatty atrophy in various muscles, pronounced in the middle part of the gluteus maximus (**a**, arrow head). The medial part of the gluteus maximus shows only beginning atrophy at T1w (**a**, arrow) together with high signal in the STIR images (**b**). T1w FS scans also display high signal after gadolinium administration (**c**). While the tensor fasciae latae muscle (wide arrow) seems to be unaffected in the T1w images, there is high signal on STIR and T1w FS scans suggesting early changes

*MRI*: standard T1w spin echo (SE) of the pelvis and thigh muscles was performed (TR = 472 ms, TE = 18 ms) and muscle fatty degenerative changes were scored according to a 5-grade modified Mercuri score (grade 0: normal, grade1: < 25%; grade 2: > 25% and < 50%; grade 3: > 50% and < 75%; grade 4: > 75%). Short TI inversion recovery (STIR) sequences of the same body regions were acquired with TR = 3240 ms, TE = 42 ms. Finally, after administration of 0.1 mmol/kg of a gadolinium-based contrast agent (Gadovist; Bayer Healthcare), a T1w fat-sat sequence was acquired (TR = 709 ms, TE = 18 ms). All MR scans were acquired axially. For STIR and T1w fat-sat contrast-enhanced (CE) images hyperintensities were again scored according to a 5-grade modified Mercuri score (grade 0: no hyperintensities, grade 1: mild; grade 2: moderate; grade 3: intermediate; grade 4: severe amount of hyperintensities). All MRIs were performed within 6 months of the FDG-PET. An experienced radiologist (AP) analysed the MRI scans. Fatty infiltration as a marker for muscular atrophy was scored based on T1w scans. Hyperintensities in T1w FS CE and STIR scans show hypervascularisation or leakage of contrast material and oedema, respectively. The following pelvic and thigh muscles were scored: autochthonous back muscle, iliopsoas, gluteus maximus, medius and minimus (lateral, mid, medial) quadriceps femoris, sartorius, obturator and pectineus. Activity and atrophy on MRI were scored as follows: from 0 to 4 (0 = no atrophy; 1 = minimal, 2 = moderate, 4 = severe). The FDG-uptake in the abovementioned muscles was visually analysed. An example for the analysis of MRI images is given in Fig. 2.

### Statistics

Statistical analysis was performed using STATISTICA 6.0 and IBM SPSS Statistics (IBM, Armonk, NY), Version 21. Data were calculated as median and interquartile range, for correlation Spearman’s coefficient of correlation was used. A *p* value < 0.05 was considered significant.

## Results

Four male patients (median age 48, interquartile range 36–55 years) participated in this explorative study. All patients were on ERT. One patient was investigated twice, before and after 1 year on ERT. All the patients demonstrated clinical improvement compared to before ERT and had either a stable (patients AB, FR) or slowly progressive (patients GH, SK) course of their disease. Details of the mutation and further clinical characteristics are indicated in Table 1. Classification [14] of disease severity of was moderate (one patient) and severe (three patients).

**Fig. 3:**
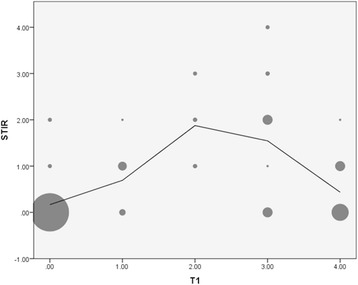
Scatterplot between the semi-quantitative assessment of T1 (atrophy) and STIR (muscle oedema): the size of the circle displays the number of muscle regions. Most muscle regions showed no atrophy (T1 = 0) and no oedema (STIR = 0). As atrophy increases so does muscular oedema, but only up to a score of T1 = 2. From there, further atrophy is associated with less oedema. Of note, some muscles showing no atrophy (T1 = 0) already exhibited oedema, possibly an early sign of affected muscle

MRI fatty infiltration in T1w images as a sign for muscular atrophy was found in all patients to varying degrees and localizations (Table 2) and correlated well with the clinical status of the patients. For all four patients, the most atrophic muscles were the intrinsic back muscles (autochthonous back muscles) and the gluteus muscles, especially the gluteus minimus. Muscles with a high degree of atrophy (score = 4) showed only low signal-intensity in STIR and T1w FS CE images (score = 0–1; score = 2 only in one case and muscle). When there was no evidence of muscular atrophy (T1w score = 0), the STIR and T1w FS CE images mostly also scored 0 in these locations. However, in two patients there were muscles without atrophic changes but with elevated STIR and T1w FS CE values. Highest score for both STIR and T1w FS CE were observed when muscular atrophy scores were between 1 and 3 (Fig. 3). Atrophy scores 0–2 positively correlated with T1w and STIR (Spearman’s *R*: 0.693, *p* < 0.05) and T1w and T1w FS CE values (Spearman’s *R*: 0.644, *p* < 0.05). In contrast, atrophy scores 2-4 demonstrated a negative correlation for T1w and STIR (Spearman’s *R*: − 0.426, *p* < 0.05) and for T1w and T1w FS CE, respectively (Spearman’s *R*: − 0.334, *p* < 0.05). There was a strong positive correlation between STIR and T1w FS CE values (Spearman’s *R*: 0.854, *p* < 0.05).

**Table 2:** Semi-quantitative results of MRI evaluation for each muscle group in Pompe disease

Muscle	T1w (Median)	T1w (IQR^a^)	STIR (Median)	STIR (IQR^a^)	T1 FS CE (Median)	T1 FS CE (IQR^a^)
Intrinsic back muscles (lower back)	3.0	3.0–3.0	0.0	0.0–3.0	0.0	0.0–3.0
Iliopsoas	2.0	0.5–3.5	1.0	0.0–2.5	1.5	0.0–2.5
Gluteus maximus (lateral)	2.0	0.5–4.0	1.0	0.0–2.0	1.5	0.5–2.0
Gluteus maximus (center)	0.0	0.0–1.5	0.0	0.0–1.5	0.0	0.0–1.5
Gluteus maximus (medial)	1.5	0.0–3.0	1.0	0.0–2.0	1.0	0.0–2.5
Gluteus medius (ventral)	2.0	1.0–3.5	1.5	1.0–2.0	2.0	1.0–3.5
Gluteus medius (dorsal)	3.5	1.5–4.0	1.0	0.0–1.5	0.5	0.0–1.5
Gluteus minimus (ventral)	4.0	3.5–4.0	0.5	0.0–1.5	0.5	0.0–1.5
Gluteus minimus (dorsal)	4.0	3.5–4.0	0.5	0.0–1.5	0.5	0.0–1.5
Rectus femoris	0.0	0.0–0.0	0.0	0.0–0.0	0.0	0.0–0.0
Sartorius	0.0	0.0–0.0	0.0	0.0–0.0	0.0	0.0–0.0
Obturator ext.	1.0	0.5–2.0	0.0	0.0–0.5	0.0	0.0–0.5
Pectineus	0.0	0.0–2.5	0.0	0.0–0.0	0.0	0.0–0.0

^a^Interquartile range

T1 images were scored according to a 5-grade score (grade 0: normal, grade 1: < 25%; grade 2: > 25% and < 50%; grade 3: > 50% and < 75%; grade 4: > 75%). STIR and T1w fat-sat contrast-enhanced (CE) images were scored according to a 5-grade score (grade 0: no hyperintensities, grade1: mild; grade 2: moderate; grade 3: intermediate; grade 4: severe amount of hyperintensities)

For all sequences, measured alterations were rather symmetrically distributed (Spearman’s *R* for atrophy = 0.875, *p* < 0.05; activity = 0.829 *p* < 0.05; T1w FS CE = 0.92 *p* < 0.05).

Assuming an alpha error of 0.05, we receive an a posteriori power of 1.000, 0.987 and 0.958 for T1, STIR and T1FSCE, respectively.

**Fig. 4:**
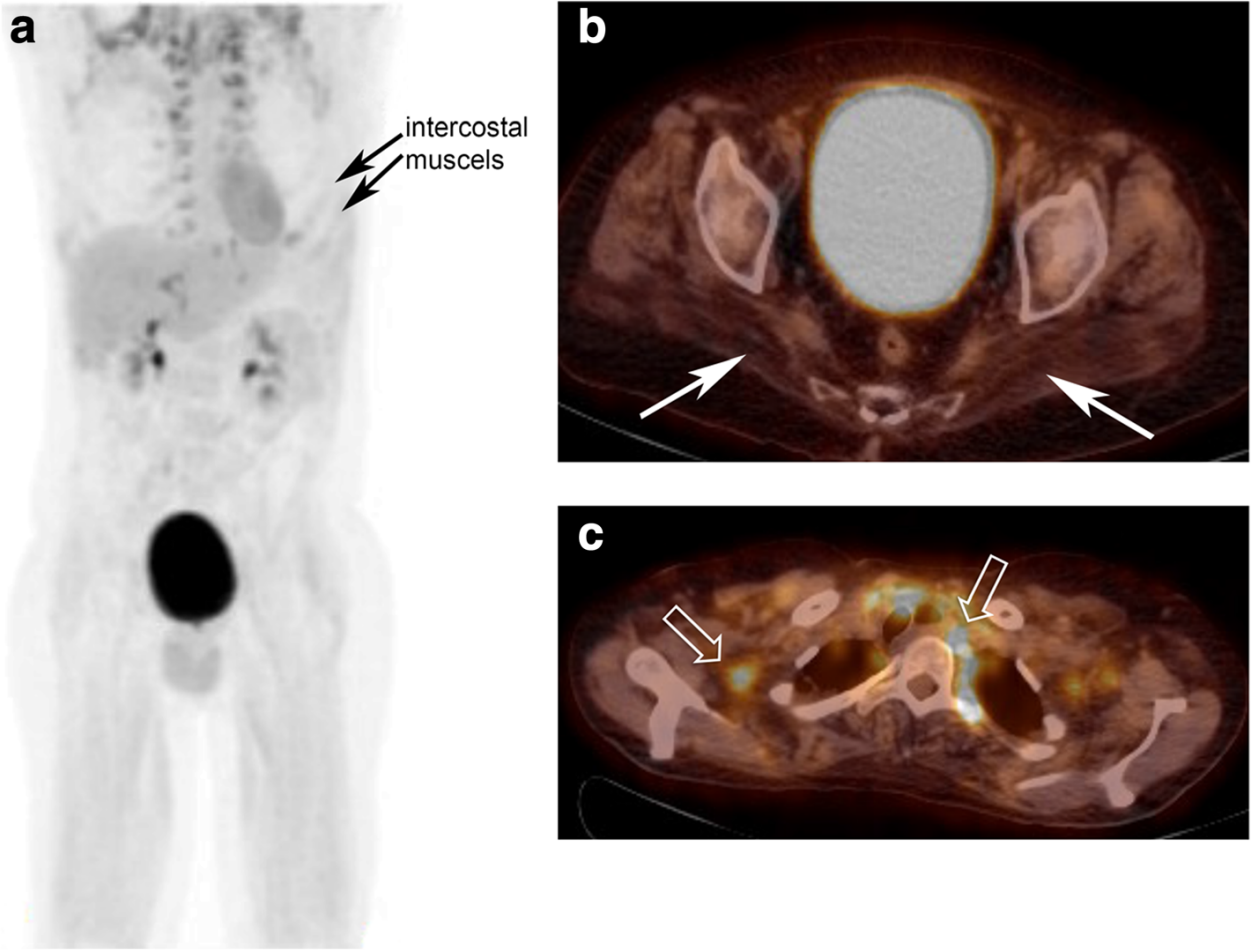
**a** Maximum intensity projection image showing the paravertebral brown fat as well as intercostal muscle. **b** Upper panel showing fused FDG PET/CT image with gluteus muscle atrophy and almost no FDG-uptake (white arrows). **c** Lower panel showing paravertebral and axillary brown fat (open arrows)

Repeated MRI in one patient demonstrated a reduction of the MRI scores at the tensor fasciae latae muscle after 1 year (Fig. 4). No other muscle group showed similar improvements.

**Fig. 5:**
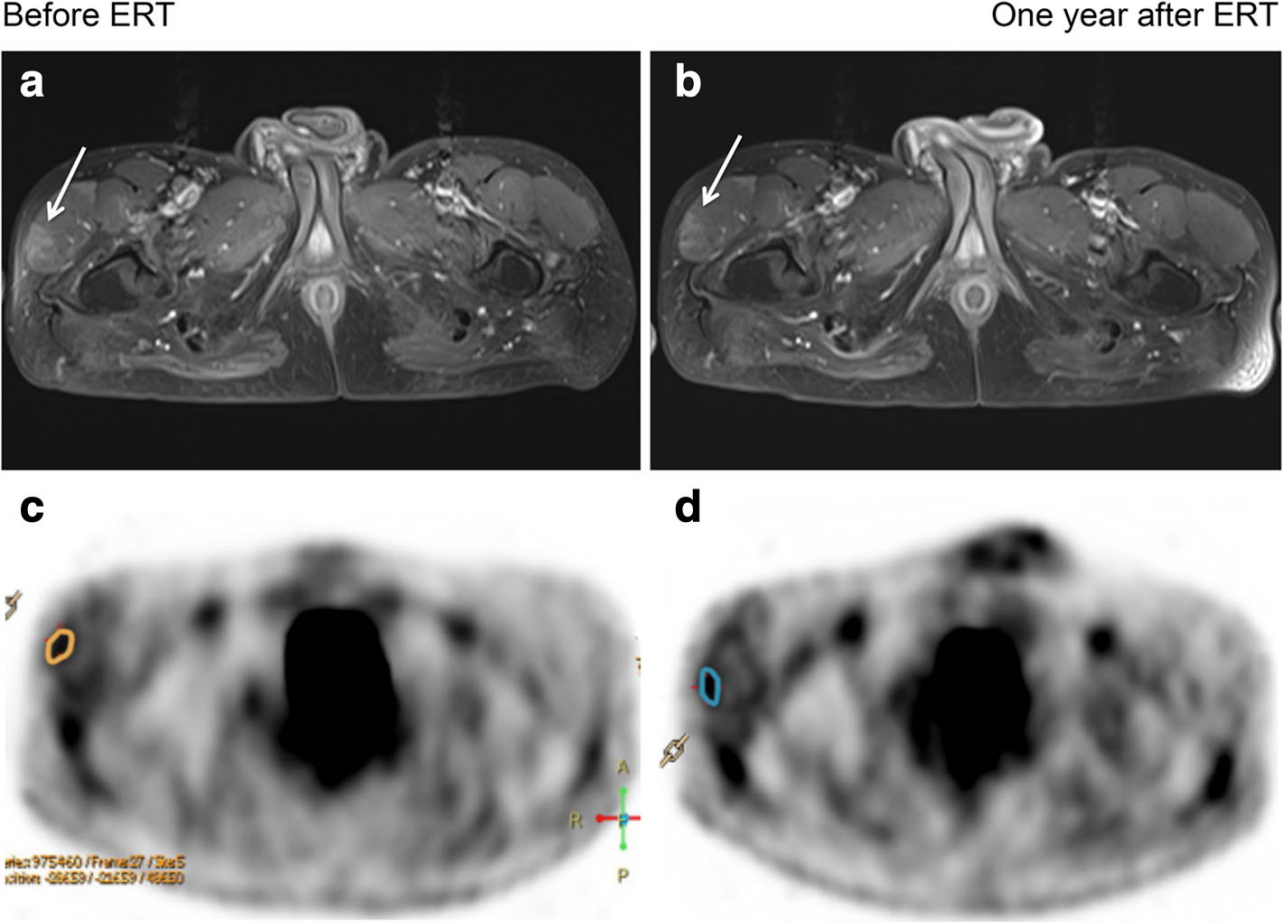
MRI (**a**, **b**) and FDG PET (**c**, **d**) before and after 1 year of ERT. MRI shows slight improvement, i.e., less hyperintensity of the tensor fasciae latae muscle (arrow) in the T1w sequence after gadolinium administration 1 year after ERT. FDG-PET show no significantly different tracer uptake in the region of interest (orange and blue lines) pre- and post-therapy

*FDG-PET/CT*. None of the muscles and/or regions of muscles demonstrating hyperintensity on MRI showed a quantifiable focally increased FDG metabolism. Nor did we detect increased FDG-metabolism in trunk or thigh muscles. Thus, dynamic PET quantification of glucose metabolism in muscles could not be calculated. Figure 5 gives an example of the results of FDG-PET.

FDG-PET was positive in one patient with respiratory failure. We observed a high uptake of FDG in the respiratory muscles. This clearly demonstrates the increased glucose metabolism due to the high muscle workload in a patient with restrictive respiratory insufficiency (Fig. 5).

## Discussion

Our pilot study confirms the usefulness of MRI to delineate affected muscles in PD [9, 12, 14–16]. The pattern of involvement was similar in patients with and without ERT [14] and disease severity and duration were correlated to quantitative MRI findings.

We analysed 26 different muscle regions of the pelvis and thighs in four different patients with late onset PD. Within individual patients, there were considerable differences in muscular atrophy of different muscles ranging from no visible atrophy (score = 0) to severe atrophy (score = 4) with the intrinsic back muscles and the gluteus minimus muscle displaying the highest scores of atrophy. We found no observable atrophy in the quadriceps femoris (rectus femoris) and sartorius muscle. Why these muscles display minor damage while others are heavily involved is a matter of debate and was also observed in earlier studies [13, 15]. Van der Ploeg et al. [13] suggests that muscle groups, like the gluteus muscles and intrinsic back muscles, performing continuous or repetitive contraction are more prone to earlier damage than muscles that are subject to relatively intermittent contraction.

STIR images revealing muscular oedema were only partly in line with the results of the T1w images showing atrophy: when muscular atrophy was not visible (score = 0) in most of the muscles analysed there was also no oedema. Oedematous changes increased with increasing atrophy, but only up to an atrophy score of 2–3 (correlation of Spearman’s *R*: 0.693). When atrophy was more prevalent (score 4) oedematous changes decreased (Spearman’s *R*: − 0.426). None of the T1w changes with a score of 4 showed an oedema score higher than 2. As a rule, similar changes as for the STIR images were noted for the T1w FS CE images in most of the muscles analysed.

The varying degree of atrophy and oedema/inflammation in different muscles in a given individual with PD—besides the already mentioned predilection of certain muscles—indicates a temporal heterogeneity of muscular changes. We hypothesize that disease manifestation in a muscle initially demonstrates only little atrophy but an increasing amount of oedema and inflammatory changes as reflected by increased STIR and T1w FS CE values. Of note, in some muscles, oedematous and inflammatory changes were visible in muscles showing no atrophy at all, consistent with results by van der Ploeg et al. [13].

After this initial stage, atrophic, oedematous and inflammatory changes seem to develop rather synchronously up to the point when inflammation and oedema are less visible, but atrophy further increases. Finally, at the end-stage of this process affected muscles display fatty infiltration as a result of atrophy but no more inflammation or oedema.

This study is the first study that used gadolinium-enhanced MR imaging for the evaluation of non-cardiac muscle involvement in late-onset PD patients. Gadolinium-based studies have shown benefits in other forms of myositis for differentiation and staging [17]. In the present study, we found a strong positive correlation between STIR and T1 FS CE images (*R* = 0.854). However, there was one significant difference between the STIR and T1 FS CE images, with the gadolinium-based T1 images showing higher values on the modified Mercuri scale. This may indicate that T1 FS CE might be more sensitive to early muscle changes in late-onset PD patients. If gadolinium studies are really necessary for early detection needs to be evaluated in further studies, especially in light of the problem of gadolinium deposition that was first described in 2014 [18].

Due to MRI limitations (claustrophobia, non-MRI compatible devices, severe respiratory disease) [10], alternative approaches like bio-impedance measurement to assess the relative proportions of fatty and muscle tissue may give a correlation between the degree of replacement of the muscle tissue with fatty tissue and the severity of the disease evaluated clinically [19], diagnosing the late phase of the disease.

In conclusion, MRI is an excellent tool to demonstrate the extent of muscle involvement in late-onset PD. MRI may even pick up early, i.e., oedematous stages of the disease. Focusing on these early signs may allow more specific training early on to set against subsequent muscular atrophy. Whether these early changes may allow prognostic predictions has yet to be demonstrated.

In well-prepared (after 6-h fast, serum glucose < 150 mg/dl and adequate rest prior to image acquisition) non-PD patients no or only faint muscular FDG uptake can be observed. We hypothesized that in PD FDG-PET/CT would delineate increased accumulation of glucose into glycogen-enriched depots and thus diagnose the early phases of the disease. In α-glucosidase-deficient muscles lysosomal glycogen breakdown and thus glucose and energy production are reduced. To compensate for reduced glucose production, increased glucose and in analogy FDG uptake may be an option in these cells. In α-glucosidase-deficient muscles intra-cytoplasmatic GLUT4 expression is increased in damaged and normal in unafflicted muscle fibres [20]. We hypothesized that this increased GLUT4 expression may correlate with an increased glucose and thus an increased FDG uptake as well.

This information may then translate in improved local physiotherapy and/or anti-inflammatory treatment. Unfortunately, no focally increased FDG accumulation in relation or unrelated to MRI-signal changes could be demonstrated. Thus, dynamic PET quantification of glycogen deposition in muscles could not be calculated. There might be multiple explanations for this result. Nutritional status, i.e., fasting or post-prandial application of the tracer may be an issue. We injected FDG in fasting patients and thus glycolysis may have prevailed glycogenesis. This is in line with our observation of an increased uptake in the respiratory muscles in the patient with severe restrictive respiratory failure. Due to the high workload of respiratory muscles glucose metabolism was increased as it was visualized in the FDG PET/CT. Again, this would indicate a rather late state of the disease, as the increased metabolic needs are correlated with a significantly increased workload on the few remaining/surviving muscles. On the other hand, with the patient supine, the energy requirement of all other muscles was low and therefore no increased glucose metabolism could be observed.

In addition, glucose uptake via GLUT4 occurs either post-prandially insulin-stimulated or during exercise [21]. However, all patients were investigated after an overnight resting phase without increased muscular energy needs. Whether post-exercise FDG may have given different results, remains to be investigated.

Alternatively, deposition of the tracer into glycogen may not have taken place at all. Glycogen is synthesized from the activated glucose-donor, uridine diphosphate glucose (UDP-glucose), catalysed by glycogen synthase, the key regulatory enzyme in glycogenesis. To be incorporated into glycogen, FDG has to be modified by glucose-6-phosphate isomerase which catalyses the second step during glycolysis. It has been suggested that the substitution of the C-2 hydroxyl moiety by ^18^F prevents this process [22]. Subsequently, FDG could possibly not be integrated into glycogen. A new tracer, 18F-N-(methyl-(2-fluoroethyl)-1H-[1,2,3]triazole-4-yl) glucosamine (^18^F-NFTG), recently developed, may be a more effective biomarker in PD. So far, 18F-NFTG PET has been used in animal tumour models to visualize tumours of the upper thorax where the rate of glycogen synthesis is cell-cycle regulated and enhanced during the non-proliferating stage of cancer cells [22]. Whether the myopathy of PD is a feasible target for integrating this new tracer for demonstrating early glycogen accumulation remains to be demonstrated.

## Conclusions

In conclusion, MRI delineated the localisation and extent of muscle atrophy in all patients. Changes in the STIR and gadolinium-based T1w sequences probably show the activity of the disease and can serve as earlier markers in the disease process than late changes revealed by fatty infiltration of atrophic muscles as measured by T1-weighted sequences.

FDG PET/CT did not demonstrate increased uptake in glycogen, neither in the early nor late phase of the tracer accumulation. A positive signal was detected by FDG PET/CT in the patient with respiratory failure. Due to the increased respiratory muscle workload, glucose utilisation and thus FDG-uptake was increased as well.

## Abbreviations

^18^F: ^18^Fluoro
^18^F-NFTG: 18F-N-(methyl-(2-fluoroethyl)-1H-[1,2,3]triazole-4-yl) glucosamine
CE: Contrast enhanced
CK: Creatinine kinase
CT: Computer tomography
ERT: Enzyme replacement therapy
FDG: 2-deoxy-2-[18]fluoro-D-glucose
FVC: Forced expiratory vital capacity
*GAA*: Acid α-glucosidase gene
Glc(4): Glcalpha1-6Glcalpha1-4glcalpha1-4Glc
GOT: Glutamat-Oxalacetet-Transaminase
GPT: Glutamat-Pyruvat-Transaminase
GS: Glycogen synthase
LDH: Lactate dehydrogenase
MBq: Megabecquerel
MRI: Magnetic resonance imaging
OMIM: Online Mendelian Inheritance in Man
PD: Pompe disease
PDFF: Proton-density fat-fraction
PET: Positron emission tomography
SD: Standard deviation
SE: Spin echo
STIR: Short TI inversion recovery
T1: T1-weighted image
TE: Echo time
TI: Inversion time
TR: Repetition time
VC: Vital capacity
Years: Years
